# Supplementary material for: Behaviour-based movement cut-off points in 3-year old children comparing wrist- with hip-worn actigraphs MW8 and GT3X
Source: PLoS One. 2025 Mar 26;20(3):e0316747. doi: 10.1371/journal.pone.0316747 (PMC11940821; doi:10.1371/journal.pone.0316747)
Supplement: S1 Table — (DOCX) [file pone.0316747.s001.docx]

# **Supplementary Information – S1 Table**

**Behaviour-based movement cut-off points in 3-year old children comparing wrist- with hip-worn actigraphs MW8 and GT3X**

Daniel Jansson^1, 2^, Rikard Westlander^3^, Jonas Sandlund^4^, Christina E. West^3^,
Magnus Domellöf^3#^, Katharina Wulff^5, 6,#,^*

Daniel Jansson^1, 2^ (ORCID ID 0000-0002-6488-0663)

Rikard Westlander^3 (^ORCID ID 0000-0002-7874-4320)

Jonas Sandlund^4^ (ORCID ID 0000-0001-5403-881)

Christina E. West^3^ (ORCID ID 0000-0001-9599-2580)

Magnus Domellöf^3^ (ORCID ID 0000-0002-0726-7029)

Katharina Wulff^5, 6^ (ORCID ID <https://orcid.org/0000-0003-2480-3329>)

^1^ Department of Community Medicine & Rehabilitation, Section of Sports Medicine, Umeå University, Umeå, Sweden

^2^Umeå School of Sport Sciences, Umeå University, Umeå, Sweden

^3^Department of Clinical Sciences, Pediatrics, Umeå University, Sweden

^4^Department of Community Medicine and Rehabilitation, Section of Physiotherapy, Umeå University, Umeå, Sweden

^5^Departments of Radiation Sciences and Molecular Biology Umeå University, Umeå, Sweden

^6^Wallenberg Centre for Molecular Medicine (WCMM), Umeå University, Umeå, Sweden

**# Joint senior authors.**

*** Corresponding author:** [Katharina.wulff@umu.se](mailto:Katharina.wulff@umu.se)

Department of Molecular Biology, 6L, Sjukhusområdet, Umeå universitet, 901 87 Umeå, Sweden.

09 January 2025

**S1 Table**. Highlighting some of the different approaches taken from sports medicine and chronobiology/sleep, with selected published examples.


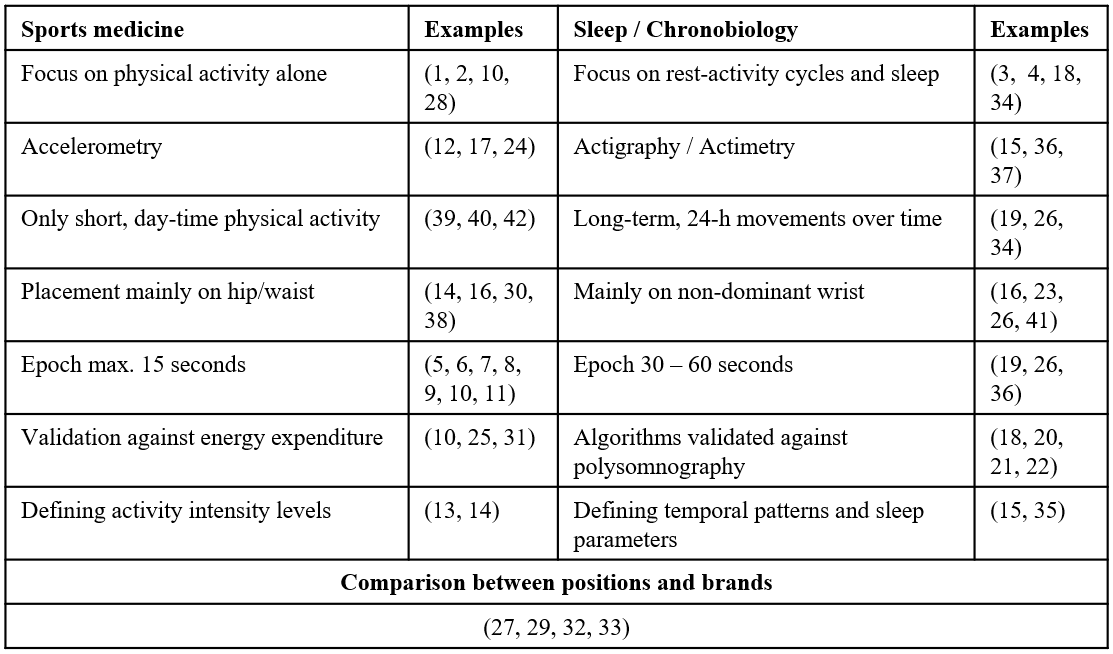


**References (Supplementary to S1 Table)**

1. Bull FC, Al-Ansari SS, Biddle S, et al. World Health Organization 2020 guidelines on physical activity and sedentary behaviour. Br J Sports Med. 2020;54(24):1451–62.

2. Møller NC, Christensen LB, Mølgaard C, Ejlerskov KT, Pfeiffer KA, Michaelsen KF. Descriptive analysis of preschool physical activity and sedentary behaviors - a cross sectional study of 3-year-olds nested in the SKOT cohort. BMC Public Health. 2017;17(1):613.

3. Hammad G, Reyt M, Beliy N, et al. pyActigraphy: Open-source python package for actigraphy data visualization and analysis. PLOS Comput Biol. 2021;17(10):e1009514.

4. Weitz M, Syed S, Hopstock LA, Morseth B, Prasad DK, Horsch A. Discrimination of sleep and wake periods from a hip-worn raw acceleration sensor using recurrent neural networks. 2022;2022.03.07.22270992. Available from: https://www.medrxiv.org/content/10.1101/2022.03.07.22270992v1.

5. Trost SG, Cliff DP, Ahmadi MN, Tuc NV, Hagenbuchner M. Sensor-enabled Activity Class Recognition in Preschoolers: Hip versus Wrist Data. Med Sci Sports Exerc. 2018;50(3):634–41.

6. van Cauwenberghe E, Labarque V, Trost SG, de Bourdeaudhuij I, Cardon G. Calibration and comparison of accelerometer cut points in preschool children. Int J Pediatr Obes IJPO Off J Int Assoc Study Obes. 2011;6(2–2):e582-589.

7. Roscoe CMP, James RS, Duncan MJ. Calibration of GENEActiv accelerometer wrist cut-points for the assessment of physical activity intensity of preschool aged children. Eur J Pediatr. 2017;176(8):1093–8.

8. Pfeiffer KA, McIver KL, Dowda M, Almeida MJCA, Pate RR. Validation and calibration of the Actical accelerometer in preschool children. Med Sci Sports Exerc. 2006;38(1):152–7.

9. Janssen X, Cliff DP, Reilly JJ, et al. Validation and calibration of the activPAL^TM^ for estimating METs and physical activity in 4-6 year olds. J Sci Med Sport. 2014;17(6):602–6.

10. Ahmadi MN, Chowdhury A, Pavey T, Trost SG. Laboratory-based and free-living algorithms for energy expenditure estimation in preschool children: A free-living evaluation. PloS One. 2020;15(5):e0233229.

11. Costa S, Barber SE, Cameron N, Clemes SA. Calibration and validation of the ActiGraph GT3X+ in 2-3 year olds. J Sci Med Sport. 2014;17(6):617–22.

12. Hislop J, Palmer N, Anand P, Aldin T. Validity of wrist worn accelerometers and comparability between hip and wrist placement sites in estimating physical activity behaviour in preschool children. Physiol Meas. 2016;37(10):1701–14.

13. Fairclough SJ, Rowlands AV, Del Pozo Cruz B, Crotti M, Foweather L, Graves LEF, Hurter L, Jones O, MacDonald M, McCann DA, Miller C, Noonan RJ, Owen MB, Rudd JR, Taylor SL, Tyler R, Boddy LM. Reference values for wrist-worn accelerometer physical activity metrics in England children and adolescents. Int J Behav Nutr Phys Act. 2023 Mar 25;20(1):35. doi: 10.1186/s12966-023-01435-z. PMID: 36964597; PMCID: PMC10039565.

14. Troiano R, McClain J, Brychta R, Chen K. Evolution of accelerometer methods for physical activity research. Br J Sports Med. 2014;48(13):1019–23.

15. Wulff K, Siegmund R. Time pattern analysis of activity-rest rhythms in families with infants using actigraphy. In: Salzarulo P, Ficca G, editors. Awakening and Sleep–Wake Cycle Across Development. John Benjamins Publishing Company; 2002. p. 149–69. [cited 2023 Aug 2 ] Available from: https://benjamins.com/catalog/aicr.38.13wul.

16. Cellini N, McDevitt EA, Mednick SC, Buman MP. Free-living cross-comparison of two wearable monitors for sleep and physical activity in healthy young adults. Physiol Behav. 2016;157:79–86.

17. Rowlands AV, Fraysse F, Catt M, et al. Comparability of measured acceleration from accelerometry-based activity monitors. Med Sci Sports Exerc. 2015;47(1):201–10.

18. Ancoli-Israel S, Cole R, Alessi C, Chambers M, Moorcroft W, Pollak CP. The role of actigraphy in the study of sleep and circadian rhythms. Sleep. 2003;26(3):342–92.

19. Falck R, Best J, Li M, Eng J, Liu-Ambrose T. Revisiting the MotionWatch8©: Calibrating Cut-Points for Measuring Physical Activity and Sedentary Behavior Among Adults With Stroke. Front Aging Neurosci. 2019;11(7):203.

20. Kushida CA, Chang A, Gadkary C, Guilleminault C, Carrillo O, Dement WC. Comparison of actigraphic, polysomnographic, and subjective assessment of sleep parameters in sleep-disordered patients. Sleep Med. 2001;2(5):389–96.

21. Cole RJ, Kripke DF, Gruen W, Mullaney DJ, Gillin JC. Automatic sleep/wake identification from wrist activity. Sleep. 1992;15(5):461–9.

22. Sadeh A, Sharkey KM, Carskadon MA. Activity-based sleep-wake identification: an empirical test of methodological issues. Sleep. 1994;17(3):201–7

23. Cyriax EF. On the Rotary Movements of the Wrist. J Anat. 1926;60(Pt 2):199–201.

24. Doherty A, Jackson D, Hammerla N, Plötz T, Olivier P, Granat MH, White T, van Hees VT, Trenell MI, Owen CG, Preece SJ, Gillions R, Sheard S, Peakman T, Brage S, Wareham NJ. Large Scale Population Assessment of Physical Activity Using Wrist Worn Accelerometers: The UK Biobank Study. PLoS One. 2017 Feb 1;12(2):e0169649. doi: 10.1371/journal.pone.0169649. PMID: 28146576; PMCID: PMC5287488.

25. Lyden K, Kozey SL, Staudenmeyer JW, Freedson PS. A comprehensive evaluation of commonly used accelerometer energy expenditure and MET prediction equations. Eur J Appl Physiol. 2011 Feb;111(2):187-201. doi: 10.1007/s00421-010-1639-8. Epub 2010 Sep 15. PMID: 20842375; PMCID: PMC3432480.

26. Wulff K, Dijk DJ, Middleton B, Foster RG, Joyce EM. Sleep and circadian rhythm disruption in schizophrenia. Br J Psychiatry. 2012 Apr;200(4):308-16. doi: 10.1192/bjp.bp.111.096321. Epub 2011 Dec 22. PMID: 22194182; PMCID: PMC3317037.

27. Mielke GI, de Almeida Mendes M, Ekelund U, Rowlands AV, Reichert FF, Crochemore-Silva I. Absolute intensity thresholds for tri-axial wrist and waist accelerometer-measured movement behaviors in adults. Scand J Med Sci Sports. 2023 Sep;33(9):1752-1764. doi: 10.1111/sms.14416. Epub 2023 Jun 12. PMID: 37306308.

28. Fairclough SJ, Rowlands AV, Taylor S, Boddy LM. Cut-point-free accelerometer metrics to assess children's physical activity: An example using the school day. Scand J Med Sci Sports. 2020 Jan;30(1):117-125. doi: 10.1111/sms.13565. Epub 2019 Oct 22. PMID: 31593604.

29. Dobell AP, Eyre ELJ, Tallis J, Chinapaw MJM, Altenburg TM, Duncan MJ. Examining accelerometer validity for estimating physical activity in pre-schoolers during free-living activity. Scand J Med Sci Sports. 2019 Oct;29(10):1618-1628. doi: 10.1111/sms.13496. Epub 2019 Jul 2. PMID: 31206785.

30. Arvidsson D, Fridolfsson J, Börjesson M, Andersen LB, Ekblom Ö, Dencker M, Brønd JC. Re-examination of accelerometer data processing and calibration for the assessment of physical activity intensity. Scand J Med Sci Sports. 2019 Oct;29(10):1442-1452. doi: 10.1111/sms.13470. Epub 2019 Jun 2. PMID: 31102474.

31. Schoffelen PFM, den Hoed M, van Breda E, Plasqui G. Test-retest variability of VO_2max_ using total-capture indirect calorimetry reveals linear relationship of VO_2_ and Power. Scand J Med Sci Sports. 2019 Feb;29(2):213-222. doi: 10.1111/sms.13324. Epub 2018 Nov 12. PMID: 30341979; PMCID: PMC7379248.

32. Lopez GA, Brønd JC, Andersen LB, Dencker M, Arvidsson D. Validation of SenseWear Armband in children, adolescents, and adults. Scand J Med Sci Sports. 2018 Feb;28(2):487-495. doi: 10.1111/sms.12920. Epub 2017 Jun 28. PMID: 28543847.

33. Hildebrand M, VAN Hees VT, Hansen BH, Ekelund U. Age group comparability of raw accelerometer output from wrist- and hip-worn monitors. Med Sci Sports Exerc. 2014 Sep;46(9):1816-24. doi: 10.1249/MSS.0000000000000289. PMID: 24887173.

34. Gössel-Symank R, Grimmer I, Korte J, Siegmund R. Actigraphic monitoring of the activity-rest behavior of preterm and full-term infants at 20 months of age. Chronobiol Int. 2004 Jul;21(4-5):661-71. doi: 10.1081/cbi-120039208. PMID: 15470961.

35. Skeldon AC, Dijk DJ, Meyer N, Wulff K. Extracting Circadian and Sleep Parameters from Longitudinal Data in Schizophrenia for the Design of Pragmatic Light Interventions. Schizophr Bull. 2022 Mar 1;48(2):447-456. doi: 10.1093/schbul/sbab124. PMID: 34757401; PMCID: PMC8886588.

36. Cosgrave J, Haines R, van Heugten-van der Kloet D, Purple R, Porcheret K, Foster R, Wulff K. The interaction between subclinical psychotic experiences, insomnia and objective measures of sleep. Schizophr Res. 2018 Mar;193:204-208. doi: 10.1016/j.schres.2017.06.058. Epub 2017 Jul 12. PMID: 28711475; PMCID: PMC5861320.

37. Meyer N, Faulkner SM, McCutcheon RA, Pillinger T, Dijk DJ, MacCabe JH. Sleep and Circadian Rhythm Disturbance in Remitted Schizophrenia and Bipolar Disorder: A Systematic Review and Meta-analysis. Schizophr Bull. 2020 Mar 10;46(5):1126–43. doi: 10.1093/schbul/sbaa024. Epub ahead of print. PMID: 32154882; PMCID: PMC7505194.

38. Wolff-Hughes DL, Bassett DR, Fitzhugh EC. Population-referenced percentiles for waist-worn accelerometer-derived total activity counts in U.S. youth: 2003 - 2006 NHANES. PLoS One. 2014 Dec 22;9(12):e115915. doi: 10.1371/journal.pone.0115915. PMID: 25531290; PMCID: PMC4274159.

39. Mwase-Vuma TW, Janssen X, Okely AD, Tremblay MS, Draper CE, Florindo AA, Tanaka C, Koh D, Hongyan G, Tang HK, Chong KH, Löf M, Hossain MS, Cross P, Chathurangana PWP, Reilly JJ. Validity of low-cost measures for global surveillance of physical activity in pre-school children: The SUNRISE validation study. J Sci Med Sport. 2022 Dec;25(12):1002-1007. doi: 10.1016/j.jsams.2022.10.003. Epub 2022 Oct 10. PMID: 36270900.

40. Moore JB, Beets MW, Brazendale K, Blair SN, Pate RR, Andersen LB, Anderssen SA, Grøntved A, Hallal PC, Kordas K, Kriemler S, Reilly JJ, Sardinha LB. Associations of Vigorous-Intensity Physical Activity with Biomarkers in Youth. Med Sci Sports Exerc. 2017 Jul;49(7):1366-1374. doi: 10.1249/MSS.0000000000001249. PMID: 28277404; PMCID: PMC5472198.

41. Danilenko KV, Stefani O, Voronin KA, et al. Wearable Light-and-Motion Dataloggers for Sleep/Wake Research: A Review. *Appl Sci*. 2022;12(22):11794.

42. Bussmann JB, van den Berg-Emons RJ. To total amount of activity….. and beyond: perspectives on measuring physical behavior. Front Psychol. 2013 Jul 22;4:463. doi: 10.3389/fpsyg.2013.00463. PMID: 23885248; PMCID: PMC3717476.
