# Supplementary material for: Behaviour-based movement cut-off points in 3-year old children comparing wrist- with hip-worn actigraphs MW8 and GT3X
Source: PLoS One. 2025 Mar 26;20(3):e0316747. doi: 10.1371/journal.pone.0316747 (PMC11940821; doi:10.1371/journal.pone.0316747)
Supplement: S2 Table — Cut-off values, sensitivity, specificity and accuracy (AUC) are reported from ‘vigorous’ to ‘motionless alert’. ‘Sedentary crafts’ and ‘Recumbent listening’ were merged into ‘Sedentary (active)’. ‘Sedentary screen time is categorised as ‘Motionless alert’. (DOCX) [file pone.0316747.s002.docx]

# **Supplementary Information – S2 Table**

**Behaviour-based movement cut-off points in 3-year old children comparing wrist- with hip-worn actigraphs MW8 and GT3X**

Daniel Jansson^1, 2^, Rikard Westlander^3^, Jonas Sandlund^4^, Christina E. West^3^,
Magnus Domellöf^3#^, Katharina Wulff^5, 6,#,^*

Daniel Jansson^1, 2^ (ORCID ID 0000-0002-6488-0663)

Rikard Westlander^3 (^ORCID ID 0000-0002-7874-4320)

Jonas Sandlund^4^ (ORCID ID 0000-0001-5403-881)

Christina E. West^3^ (ORCID ID 0000-0001-9599-2580)

Magnus Domellöf^3^ (ORCID ID 0000-0002-0726-7029)

Katharina Wulff^5, 6^ (ORCID ID <https://orcid.org/0000-0003-2480-3329>)

^1^ Department of Community Medicine & Rehabilitation, Section of Sports Medicine, Umeå University, Umeå, Sweden

^2^Umeå School of Sport Sciences, Umeå University, Umeå, Sweden

^3^Department of Clinical Sciences, Pediatrics, Umeå University, Sweden

^4^Department of Community Medicine and Rehabilitation, Section of Physiotherapy, Umeå University, Umeå, Sweden

^5^Departments of Radiation Sciences and Molecular Biology Umeå University, Umeå, Sweden

^6^Wallenberg Centre for Molecular Medicine (WCMM), Umeå University, Umeå, Sweden

**# Joint senior authors.**

*** Corresponding author:** [Katharina.wulff@umu.se](mailto:Katharina.wulff@umu.se)

Department of Molecular Biology, 6L, Sjukhusområdet, Umeå universitet, 901 87 Umeå, Sweden.

09 January 2025

**ROC curve results of One-vs-One ROC**

The first round of ROC analysis was performed based on the One-vs-One (OvO) scheme **(S2 Table)**. Cut-off points were derived from pairwise combination of counts of adjacent behavioural activities. The behavioural activities were sorted from highest to lowest expected intensities to evaluate the accuracy of separating thresholds stepwise from high physical behaviour (sprinting) to motionless-alert (watching cartoons), see schematic (**Fig. 2a** in the main text).

**S2 Table.** Receiver operating characteristics curve (ROC) analysis in One-vs-One scheme, comparing pairwise combination of adjacent behavioural classes as described in **Fig. 2a.** Cut-off values, sensitivity, specificity and accuracy (AUC) are reported from ‘vigorous’ to ‘motionless alert’. ‘Sedentary crafts’ and ‘Recumbent listening’ were merged into ‘Sedentary (active)’. ‘Sedentary screen time is categorised as ‘Motionless alert’.

|  | **Wrist- worn MotionWatch 8** | | | | | **Wrist-worn ActiGraph (GT3X)** | | | | |
| --- | --- | --- | --- | --- | --- | --- | --- | --- | --- | --- |
|  | Cut point value (counts) | Sensitivity (%) | Specificity (%) | AUC  (95% CI) |  | Cut point value (counts) | Sensitivity (%) | Specificity (%) | AUC  (95% CI) |  |
| Vigorous activity^1^ | >1040 | 80.4 | 87.6 | 0.91  (0.89 to 0.93)* |  | ≥6677 | 81.7 | 86.0 | 0.90  (0.88 to 0.92) * |  |
| Moderate activity^2^ | >445 | 76.6 | 87.4 | 0.88  (0.86 to 0.90) * |  | >3402 | 71.9 | 90.6 | 0.87  (0.85 to 0.89) * |  |
| Light activity^3^ | >154 | 84.9 | 70.7 | 0.85  (0.82 to 0.87) * |  | >1760 | 79.0 | 77.2 | 0.84  (0.82 to 0.86) * |  |
| Sedentary (active) | 24.5-154 | NA | NA | NA |  | 933-1760 | NA | NA | NA |  |
| Motionless alert^4^ | <24.5 | 76.8 | 53.4 | 0.69  (0.66 to 0.71) * |  | <933 | 54.0 | 80 | 0.71  (0.69 to 0.71) |  |
|  | **Hip-worn MotionWatch 8** | | | | | **Hip-worn ActiGraph (GT3X)** | | | | |
|  | Cut point value (counts) | Sensitivity (%) | Specificity (%) | AUC  (95% CI) |  | Cut point value (counts) | Sensitivity (%) | Specificity (%) | AUC  (95% CI) |  |
| Vigorous activity^1^ | >875 | 82.9 | 92.2 | 0.92  (0.90 to 0.93) * |  | >1866 | 77.3 | 46.2 | 0.62  (0.59 to 0.65) * |  |
| Moderate activity^2^ | >230 | 92.2 | 94.2 | 0.98  (0.97 to 0.99) * |  | >1655 | 66.1 | 87.8 | 0.84  (0.81 to 0.86) * |  |
| Light activity^3^ | >13 | 83.3 | 71.0 | 0.84  (0.81 to 0.86) * |  | >442 | 86.9 | 81.3 | 0.90  (0.88 to 0.91) * |  |
| Sedentary (active) | 2-13 | NA | NA | NA |  | 16-442 | NA | NA | NA |  |
| Motionless alert^4^ | <2 | 47.6 | 69.6 | 0.59  (0.56 to 0.620) |  | <16 | 76.7 | 58.4 | 0.68  (0.65to 0.70)* | - |

*p< 0.001

^1^Vigorous activity versus moderate activity

^2^Moderate activity versus light activity

^3^Light activity versus merged sedentary crafts and recumbent listening

^4^Merged sedentary crafts and recumbent listening versus sedentary screen time

The OvO ROC-AUC analysis returned cut-offs of good to excellent probability of correctly identifying adjacent ‘*physically mobile’* classes: ‘vigorous’, ‘moderate’ and ‘light’ activity (**Tab. S2**). However, the OvO ROC-AUC analysis returned cut-offs of fair probability of correctly identifying adjacent ‘*physically stationary’* behaviours for the GT3X devices and cut-offs of poor probability for the MW8 devices, regardless of position (**Tab. S2**). The large interquartile ranges and great overlap between classes among ’*physically stationary’* activities, especially ‘sedentary screen time’ and ‘recumbent listening’, likely precluded reliably excluding counts not belonging to their respective class (see **Fig. 4,** in the main text).
