# Supplementary material for: Behaviour-based movement cut-off points in 3-year old children comparing wrist- with hip-worn actigraphs MW8 and GT3X
Source: PLoS One. 2025 Mar 26;20(3):e0316747. doi: 10.1371/journal.pone.0316747 (PMC11940821; doi:10.1371/journal.pone.0316747)
Supplement: S7 Fig — OvR ROC curves illustrating the results in Tables 3 and 4 in the main text for: A) vigorous physical activity (VPA), B) moderate-vigorous physical activity (MVPA), C) light moderate vigorous physical activity (LMVPA) and D) motionless-alert (MOA). (DOCX) [file pone.0316747.s007.docx]

# **Supplementary Information – S7 Figure**

**Behaviour-based movement cut-off points in 3-year old children comparing wrist- with hip-worn actigraphs MW8 and GT3X**

Daniel Jansson^1, 2^, Rikard Westlander^3^, Jonas Sandlund^4^, Christina E. West^3^,
Magnus Domellöf^3#^, Katharina Wulff^5, 6,#,^*

Daniel Jansson^1, 2^ (ORCID ID 0000-0002-6488-0663)

Rikard Westlander^3 (^ORCID ID 0000-0002-7874-4320)

Jonas Sandlund^4^ (ORCID ID 0000-0001-5403-881)

Christina E. West^3^ (ORCID ID 0000-0001-9599-2580)

Magnus Domellöf^3^ (ORCID ID 0000-0002-0726-7029)

Katharina Wulff^5, 6^ (ORCID ID <https://orcid.org/0000-0003-2480-3329>)

^1^ Department of Community Medicine & Rehabilitation, Section of Sports Medicine, Umeå University, Umeå, Sweden

^2^Umeå School of Sport Sciences, Umeå University, Umeå, Sweden

^3^Department of Clinical Sciences, Pediatrics, Umeå University, Sweden

^4^Department of Community Medicine and Rehabilitation, Section of Physiotherapy, Umeå University, Umeå, Sweden

^5^Departments of Radiation Sciences and Molecular Biology Umeå University, Umeå, Sweden

^6^Wallenberg Centre for Molecular Medicine (WCMM), Umeå University, Umeå, Sweden

**# Joint senior authors.**

*** Corresponding author:** [Katharina.wulff@umu.se](mailto:Katharina.wulff@umu.se)

Department of Molecular Biology, 6L, Sjukhusområdet, Umeå universitet, 901 87 Umeå, Sweden.

09 January 2025

**Figures of One-versus-Rest ROC curves supplementing**

**the results in the main text**


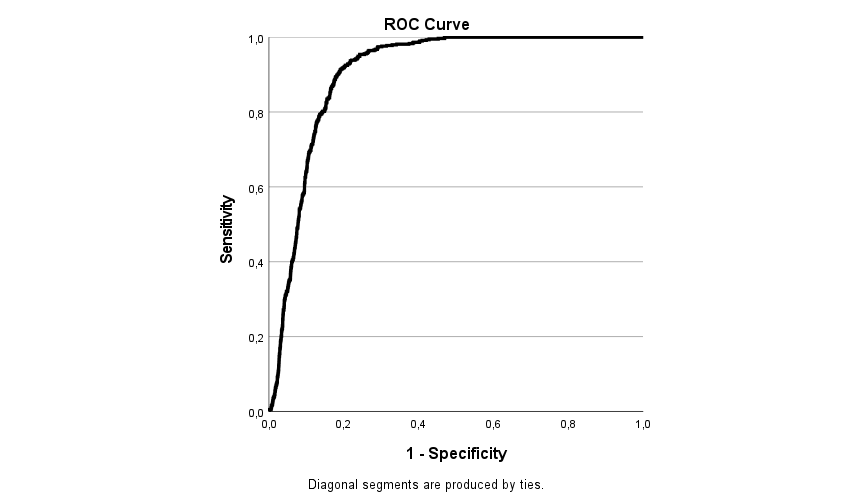

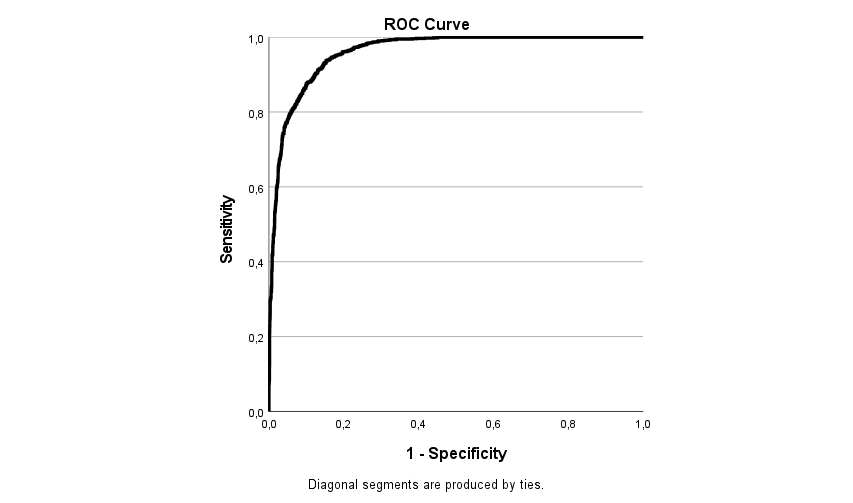

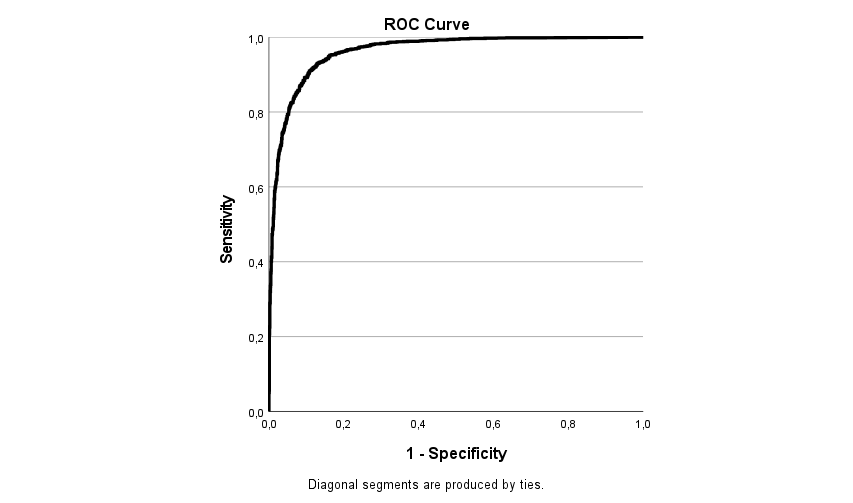

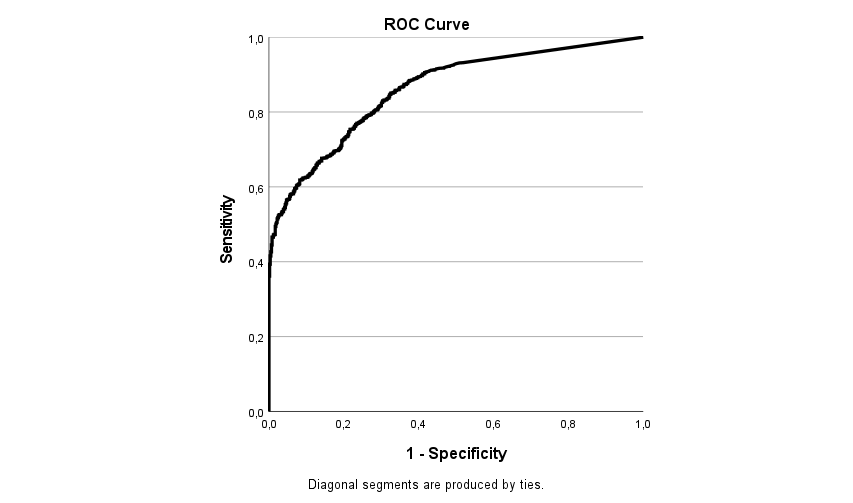


A.

B.

D.

C.

**S7 Fig.** **ROC curves for hip-worn GT3X.** OvR ROC curves illustrating the results in table 3 and table 4 in the main text for: **A**) vigorous physical activity (VPA), **B)** moderate-vigorous physical activity (MVPA), **C)** light moderate vigorous physical activity (LMVPA) and **D**) motionless-alert (MOA).
